# Cucumber *Phospholipase D alpha* gene overexpression in tobacco enhanced drought stress tolerance by regulating stomatal closure and lipid peroxidation

**DOI:** 10.1186/s12870-018-1592-y

**Published:** 2018-12-14

**Authors:** Tuo Ji, Shuzhen Li, Lujun Li, Meili Huang, Xiufeng Wang, Min Wei, Qinghua Shi, Yan Li, Biao Gong, Fengjuan Yang

**Affiliations:** 1 State Key Laboratory of Crop Biology, College of Horticulture Science and Engineering, Shandong Agricultural University, Tai’an, 271018 People’s Republic of China; 2 Key Laboratory of Biology and Genetic Improvement of Horticultural Crops (Huanghuai Region), Ministry of Agriculture, Tai’an, 271018 People’s Republic of China

**Keywords:** *CsPLDα*, Transgenic tobacco, Phosphatidic acid, Drought stress, Abscisic acid, Stomatal closure, Lipid peroxidation

## Abstract

**Background:**

Plant phospholipase D (PLD), which can hydrolyze membrane phospholipids to produce phosphatidic acid (PA), a secondary signaling molecule, has been proposed to function in diverse plant stress responses. Both PLD and PA play key roles in plant growth, development, and cellular processes. PLD was suggested to mediate the regulation of stomatal movements by abscisic acid (ABA) as a response to water deficit. In this research, we characterized the roles of the cucumber *phospholipase D alpha* gene (*CsPLDα*, GenBank accession number EF363796) in the growth and tolerance of transgenic tobacco (*Nicotiana tabacum*) to drought stress.

**Results:**

The *CsPLDα* overexpression in tobacco lines correlated with the ABA synthesis and metabolism, regulated the rapid stomatal closure in drought stress, and reduced the water loss. The *NtNCED1* expression levels in the transgenic lines and wild type (WT) were sharply up-regulated after 16 days of drought stress compared with those before treatment, and the expression level in the transgenic lines was significantly higher than that in the WT. The *NtAOG* expression level evidently improved after 8 and 16 days compared with that at 0 day of treatment and was significantly lower in the transgenic lines than in the WT. The ABA content in the transgenic lines was significantly higher than that in the WT. The Cs*PLDα* overexpression could increase the osmolyte content and reduce the ion leakage. The proline, soluble sugar, and soluble protein contents significantly increased. By contrast, the electrolytic leakage and malondialdehyde accumulation in leaves significantly decreased. The shoot and root fresh and dry weights of the overexpression lines significantly increased. These results indicated that a significant correlation between *CsPLDα* overexpression and improved resistance to water deficit.

**Conclusions:**

The plants with overexpressed *CsPLDα* exhibited lower water loss, higher leaf relative water content, and heavier fresh and dry matter accumulation than the WT. We proposed that *CsPLDα* was involved in the ABA-dependent pathway in mediating the stomatal closure and preventing the elevation of intracellular solute potential.

**Electronic supplementary material:**

The online version of this article (10.1186/s12870-018-1592-y) contains supplementary material, which is available to authorized users.

## Background

Environmental stresses trigger a wide variety of plant responses, and drought stress is one of the most adverse factors to plant growth and productivity [1, 2]. Drought stress can increase reactive oxygen species (ROS) generation, increase pyruvic acid content, decrease ascorbic acid content, induce lipid peroxidation injury, and cause irreversible damage, which leads to death [3]. Plants form a complex regulatory mechanism to adapt or resist water deficit during prolonged evolution. Before drought occurs, plants accelerate tissue maturation to effectively avoid damage. Plants develop a strong root system by closing stomata to reduce water loss [4]. External stimuli can be identified by plant cell membranes or osmosensors, leading to the production of secondary messengers that can be transported within the cell [5]. The secondary messengers can adjust the phosphorylation status of downstream proteins by regulating the activity of intracellular protein kinase, invoking the activity of transcription factors, mediating the expression of target genes in the nucleus [6]. These processes affect the plant morphogenesis, transformation of carbon metabolic pathways, hormone synthesis, ROS balance, and osmolyte accumulation, eventually enhancing plant resistance to stress [7, 8]. When plants sense a drought stress signal, intracellular signaling molecules, such as secondary messengers, are transmitted inside the cell. The signal transduction activates the abscisic acid (ABA)-dependent/independent pathways, which allow water deficit resistance [9]. At least three ABA-dependent pathways exist in plants, and the transcription factors that regulate these pathways include myeloblastosis, NAC (NAM/ATAF1/2/CUC2), and others [10, 11]. ABA-independent pathway-related genes mainly include those that possess dehydrating response components in their promoter regions and transcription factors that combine with dehydration-responsive element/C-repeat components [12]. Therefore, differences in ABA accumulation in plants induce different pathways in response to water deficit. Most of the genes related to ABA synthesis and degradation pathways including *ZEP*/*ABA1*, *NCED*, *SDR*/*ABA2*, *LOS5*/*ABA3*, *AAO3*/*ABA5*, *CYP707A*, and *AOG*, have been cloned and studied [13–15]. One study suggested that the *SDR* gene is not affected by the drought stress signal [16]. Hence, ABA plays a vital role in the water deficit response mechanism of plants. ABA participates in the plant response to drought stress by controlling the opening and closing of plant stomata [17, 18]. The stress hormone ABA and elevated CO_2_ levels activate complex signaling pathways that are mediated by kinases/phosphatases, secondary messengers, and ion channel regulation in guard cells [19].

Some transcription factor genes respond to drought stress signal, but mainly two kinds of protein participate in the water deficit response: the regulatory and function proteins. The regulatory proteins include protein kinases, transcription factors, and phospholipases. These proteins are involved in the signal transduction of drought stress mainly by adjusting other signaling molecules. The function proteins include the LEA-like protein, molecular chaperones, osmolyte synthetases, transporters, and ROS detoxification protein enzymes. They are directly involved in the drought stress response and repair process [6]. Among these proteins, the plant phospholipase D (PLD) family and the phosphatidic acid (PA) they produce function in drought stress responses [20–23]. PLD exhibits the dual function of membrane degradation and signal transduction [21]. As a lipid-hydrolyzing enzyme, PLD hydrolyzes membrane phospholipids to produce PA and exhibits increased activity under dehydration and hyperosmotic conditions [24, 25]. The produced PA acts as a secondary messenger, amplifying the signal to possibly mitigate stress injury; thus, it mainly functions in stress injury rather than in membrane degradation in some cases [26]. The detrimental effects of drought stress are prevented by minimizing cuticular water loss and maximizing water uptake [27]. *PLDα1* mediates ABA regulation, which controls stomatal closure and decreases transpirational water loss in response to water deficits [28, 29]. *Arabidopsis* with abrogated *PLDα1* is insensitive to ABA-mediated stomatal closure and exhibits more water loss than that of the wild type (WT) [28], whereas *Arabidopsis* with overexpressed *PLDα1* loses less water than the WT [30]. On one hand, *PLDα1*-produced PA binds to ABI1 protein phosphatase 2C, and this interaction may tether ABI1 to the plasma membrane, impede its negative function on ABA response, and enhance ABA-promoted stomatal closure [28, 29]. On the other hand, *PLDα1* interacts with the Gα subunit of heterotrimeric G protein to mediate stomatal opening inhibition by ABA [29, 31]. PA also binds to NADPH oxidase and stimulates its activity to promote ROS or NO production in ABA-mediated stomatal closure [32]. Additional studies are needed in order to further confirm these results.

Our previous study showed that the cucumber *phospholipase D alpha* gene (*CsPLDα*) was involved in the response to hyperosmotic stress, and the overexpression of *CsPLDα* in tobacco could enhance the tolerance to high salinity, polyethylene glycol and ABA treatments, which was proved in seed germination and seedling condition [33]. Furthermore, the *CsPLDα*-produced PA participated in the salt response by congesting osmolytes, balancing Na^+^–K^+^ ratio and eliminating the accumulation of ROS indirectly [34]. In the current study, we found that *CsPLDα*-produced PA could do more work in drought stress response, especially in promoting ABA-mediated stomatal closure, balancing osmolytes, and stabilizing the membrane system, which could keep more water in plants. Therefore, these findings suggested that *CsPLDα* contributed significantly to drought stress in plants.

## Results

### *NtPLDα1* and *NtNAC072* expression under drought stress

**Fig. 1:**
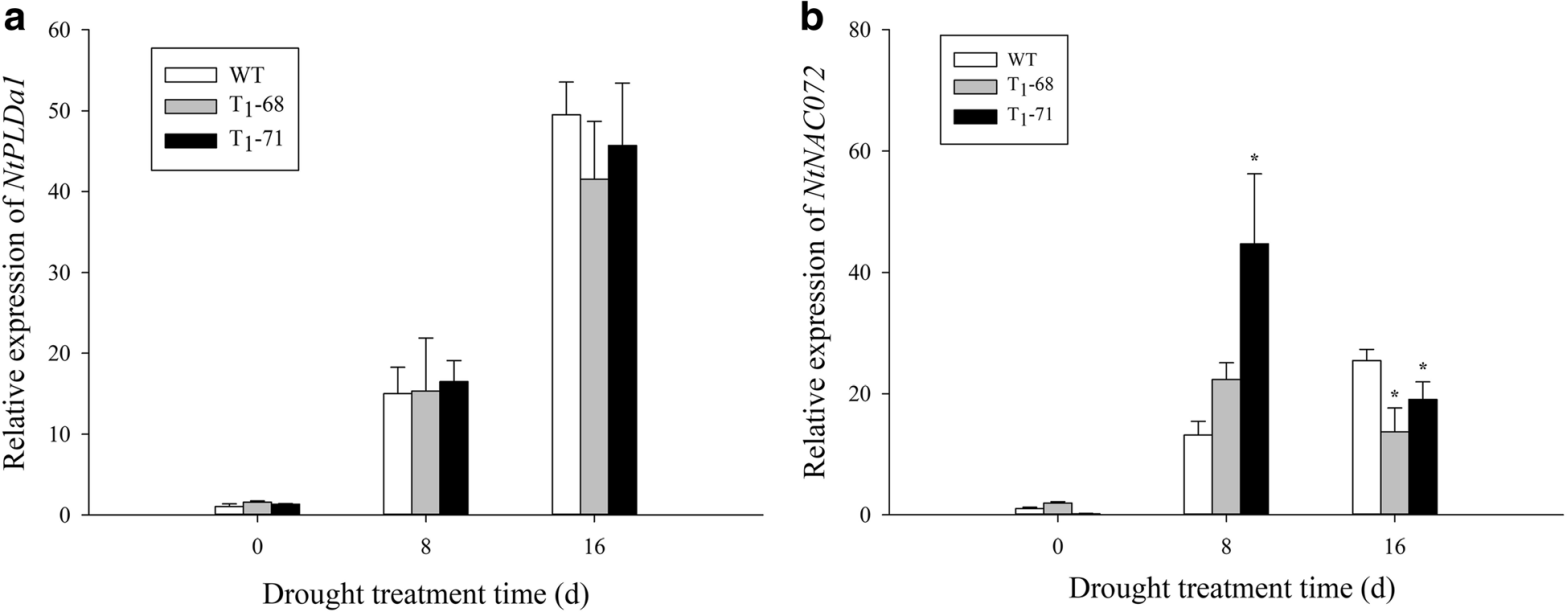
Effects of drought stress on the expression level of endogenous *NtPLDα1*(**a**) and *NtNAC072* (**b**) in leaves of both wild type (WT) and transgenic tobacco after treatment of 0, 8 and 16 d. Values are means ± SD (*n* = 3). * and ** Significant at *P* < 0.05 and *P* < 0.01 compared with WT, respectively

To determine the involvement of endogenous *NtPLDα1* in drought responses, the *NtPLDα1* expression levels in the leaves of the WT and transgenic plants were measured (Fig. 1a). No difference in the *NtPLDα1* expression was observed between the WT and transgenic plants before treatment (0 day) and after 8 and 16 days of drought stress treatment. However, the expression level of *NtNAC072*, a drought-induced marker gene, increased after 8 days of treatment and was higher in the transgenic lines than in the WT. The *NtNAC072* expression level in the ‘T_1_–71’ leaves was 2.39 times higher than that in the WT. The *NtNAC072* expression level decreased significantly after 16 days (*P* < 0.05) (Fig. 1b). Thus, the *CsPLDα* overexpression could enhance the sensitivity of plants to drought stress.

### *CsPLDα* mediated the plant response to ABA synthesis and metabolism, stomatal closure, and water loss

**Fig. 2:**
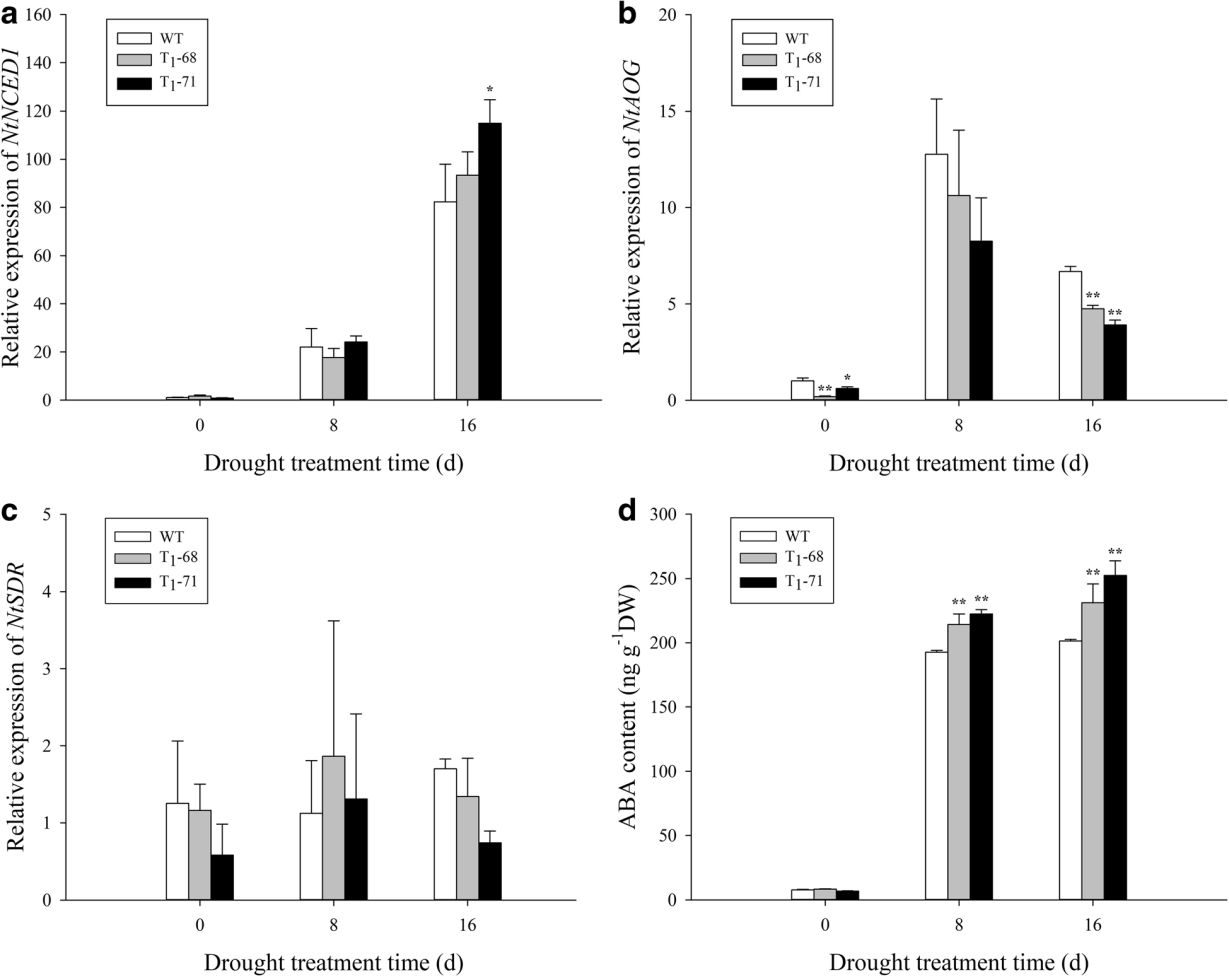
Effects of drought stress on the relative expression of *NtNCED1*(**a**), *NtAOG* (**b**), *NtSDR* (**c**) and the ABA content (**d**) in leaves of both wild type (WT) and transgenic tobacco after treatment of 0, 8 and 16 d. Values are means ± SD (*n* = 3). * and ** Significant at *P* < 0.05 and *P* < 0.01 compared with WT, respectively

To determine the involvement of *CsPLDα* in modulating the ABA synthesis and metabolism during water deficit, RT-PCR was conducted to analyze the expression levels of ABA-related genes (Fig. 2), including two key genes in the ABA synthesis and metabolism pathway, namely, *NtNCED1* and *NtAOG*. *NtNCED1* is a rate-limiting enzyme in ABA metabolism, whereas *NtAOG* controls the ABA metabolism. As shown in Fig. 2, the *NtNCED1* expression level increased approximately 100 times in all group after 16 days of stress compared with that before treatment, and was significantly higher by 39.8% in ‘T_1_–71’ and 13.5% in ‘T_1_–68’ (*P* < 0.05) compared with that in the WT after 16 days of stress (Fig. 2a). The *NtAOG* expression also evidently enhanced at 8 and 16 days after treatment compared with that at 0 day of treatment, whereas the transgenic lines exhibited a significantly lower expression than that of the WT (Fig. 2b). No difference in *NtSDR* expression was observed between the transgenic lines and WT at all treatment times (Fig. 2c). The ABA content in the tobacco plants was lower before treatment (0 day) and increased almost 30 times at 8 days after the drought stress in both the OE lines and WT. The ABA contents in the leaves of ‘T_1_–68’ and ‘T_1_–71’ transgenic plants were significantly higher by 11.2 and 15.5%, respectively (*P* < 0.01). The same significant difference was maintained at 16 days after the water deficit (Fig. 2d).

**Fig. 3:**
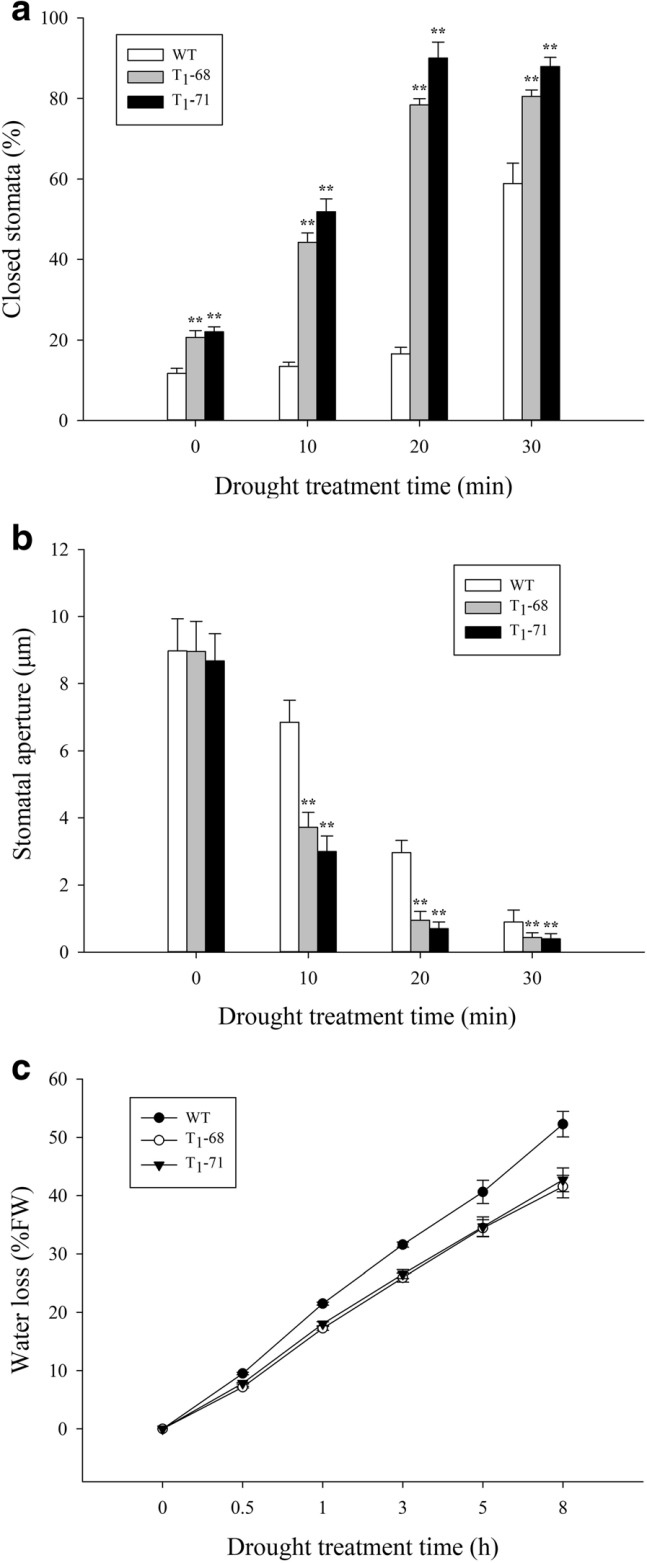
Effects of drought stress on stomatal closure percentage (**a**), stomatal aperture (**b**) after detachment on 0, 10, 20 and 30 min and water loss (**c**) after detachment on 0, 0.5, 1, 3, 5 and 8 h in leaves of both wild type (WT) and transgenic tobacco. Values are means ± SD (*n* = 15 in (**a**, **b**) and *n* = 3 in **c**). * and ** Significant at *P* < 0.05 and *P* < 0.01 compared with WT, respectively

The role of *CsPLDα* in the ABA-promoted stomatal closure was also studied (Fig. 3). When the leaves were detached at 0 min, almost 90% of the stomata were opened in both the OE lines and WT, and the percentages of closed stomata were similar in the OE lines and WT. The dehydration of the detached leaves promoted stomatal closure faster in the OE lines than that in the WT. The percentages of closed stomata of the ‘T_1_–68’ and ‘T_1_–71’ transgenic plants were 41.2 and 51.9%, respectively. The percentage of closed stomata was only 13.4% in the WT after 10 min of detachment. The percentage of closed stomata increased up to 78.4 and 90.0% in ‘T_1_–68’ and ‘T_1_–71’ at 20 min of detachment, respectively. Almost no difference between OE lines was observed at 30 min of detachment compared to at 20 min of detachment. However, the percentage of closed stomata in the WT was 58.9% after 30 min of detachment; such increase was faster than that after 20 min of detachment (Fig. 3a). The apertures of the remaining opened stomata were measured, and an evident difference was observed between the OE plants and WT. The stomatal apertures of the ‘T_1_–68’ and ‘T_1_–71’ transgenic plants were 3.7 and 3.0 μm, respectively, but stomatal aperture of the WT was 6.6 μm after 10 min of detachment. The stomatal apertures of ‘T_1_–68’ and ‘T_1_–71’ significantly declined by 2.79 and 3.08 times after 20 min of detachment compared with those after 0 min of detachment. The stomatal aperture remained in the WT at 3.0 μm (Fig. 3b). The water loss of the ‘T_1_–68’ and ‘T_1_–71’ transgenic plants exhibited no obvious difference, but that of the WT was higher than that in the OE lines during 0–8 h of treatment. After 8 h of treatment, the water loss of the WT plant was significantly higher by 25.8 and 22.4% than that of ‘T_1_–68’ and ‘T_1_–71’, respectively (Fig. 3c). These findings suggested that *CsPLDα* played an important role in promoting stomatal closure.

### FW and DW under drought stress

**Fig. 4:**
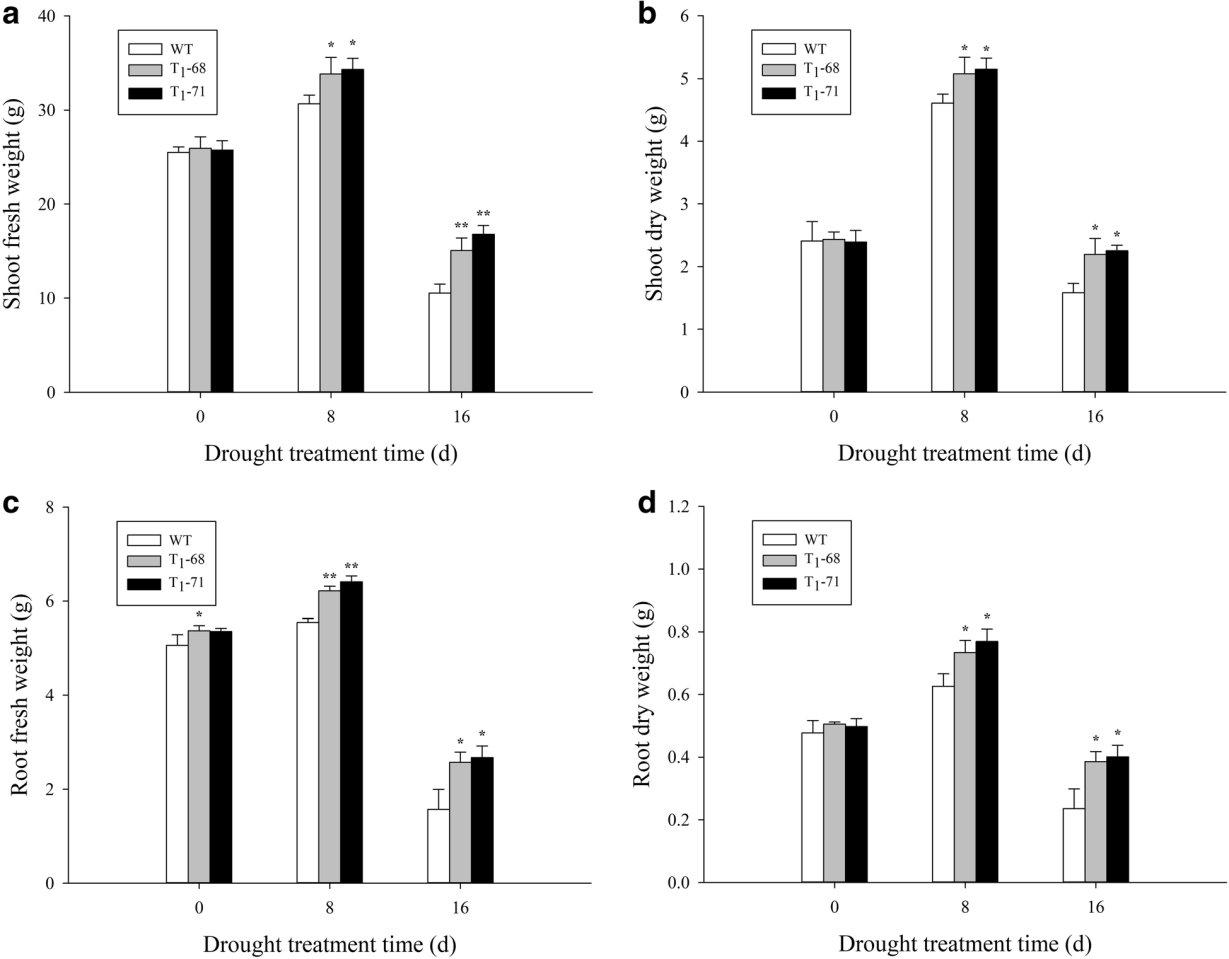
Shoots and roots fresh weights (**a**, **c**) and dry weights (**b**, **d**) of wild type (WT) and transgenic tobacco seedlings were measured after drought stress treatment of 0, 8 and 16 d, separately. Values are means ± SD (*n* = 3). * and ** Significant at *P* < 0.05 and *P* < 0.01 compared with WT, respectively

As shown in Fig. 4, no significant difference between the transgenic plants and WT was observed under normal conditions (0 d). A remarkable accumulation of shoots and roots occurred after 8 days of treatment. However, the shoot and root growths of both the WT and transgenic seedlings were severely suppressed after 16 days of exposure to drought stress. In particular, the growth of the WT seedlings was significantly inhibited (*P* < 0.05). The FWs (Fig. 4a) and DWs (Fig. 4b) of the WT, ‘T_1_–68’, and ‘T_1_–71’ shoots at 16 days of treatment were significantly lower by 58.7, 41.9, and 34.8% and by 34.3, 10.0, and 5.8%, respectively, compared with those at 0 day. The FWs (Fig. 4c) and DWs (Fig. 4d) of the WT, ‘T_1_–68’, and ‘T_1_–71’ roots at 16 days of treatment were significantly lower by 69.0, 51.8, and 50.0% and by 50.5, 23.5, and 19.2%, respectively, compared with those at 0 day. After 16 days of treatment, the shoot FWs and DWs of the ‘T_1_–68’ transgenic plants increased by 43.1 and 38.6%, respectively, and those of the ‘T_1_–71’ transgenic plants increased by 59.4 and 42.3%, respectively, compared with those of the WT. After 16 days of treatment, the root FWs and DWs of the ‘T_1_–68’ transgenic plants increased by 63.8 and 63.6%, respectively, and those of the ‘T_1_–71’ transgenic plants increased by 70.2 and 70.1%, respectively, compared with those of the WT. Thus, the transgenic tobacco plants exhibited improved drought stress resistance, and *CsPLDα* may play an important role in alleviating the stress damage caused by heavy drought.

### EL and MDA content under drought stress

**Fig. 5:**
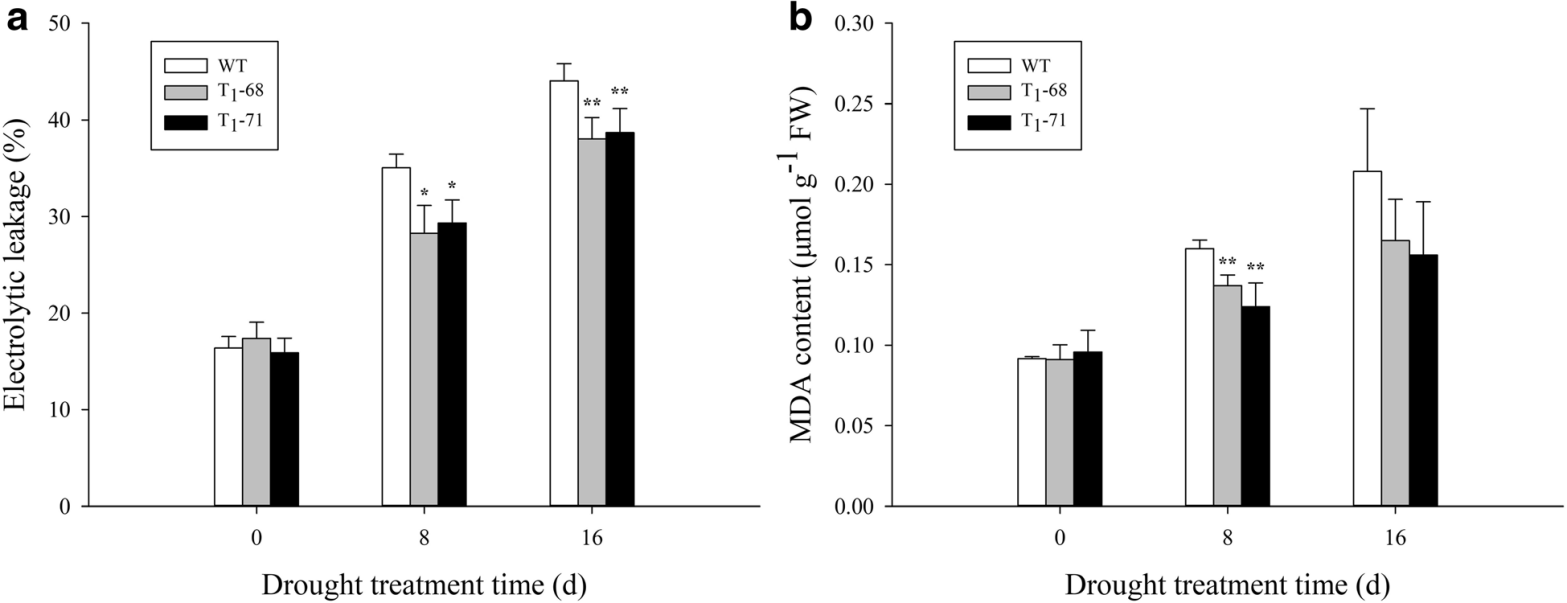
Effects of drought stress on electrolytic leakage (EL) (**a**) and malondialdehyde (MDA) content (**b**) in leaves of both wild type (WT) and transgenic tobacco after treatment of 0, 8 and 16 d. Values are means ± SD (*n* = 3). * and ** Significant at *P* < 0.05 and *P* < 0.01 compared with WT, respectively

Figure 5 shows that under normal conditions (0 day), the EL and MDA content of the leaves of the transgenic and WT plants exhibited no significant difference. The EL and MDA content increased compared with those at 0 day with increasing drought time but were significantly lower in the transgenic lines than in the WT (*P* < 0.05). The EL of the WT, ‘T_1_–68’, and ‘T-71’ increased by 114.0, 62.7, and 84.5% and by 168.9, 118.9, and 143.6% (Fig. 5a) after 8 and 16 days of treatment, respectively, compared with those at 0 day of treatment. The MDA content of the WT, ‘T_1_–68’, and ‘T-71’ increased by 74.1, 49.9, and 29.1% and by 127.0, 81.2, and 62.7% (Fig. 5b) after 8 and 16 days of treatment, respectively, compared with those at 0 day of treatment. These results indicated that *CsPLDα* played an important role in maintaining membrane system stability.

### Soluble sugar, proline, and soluble protein contents under drought stress

**Fig. 6:**
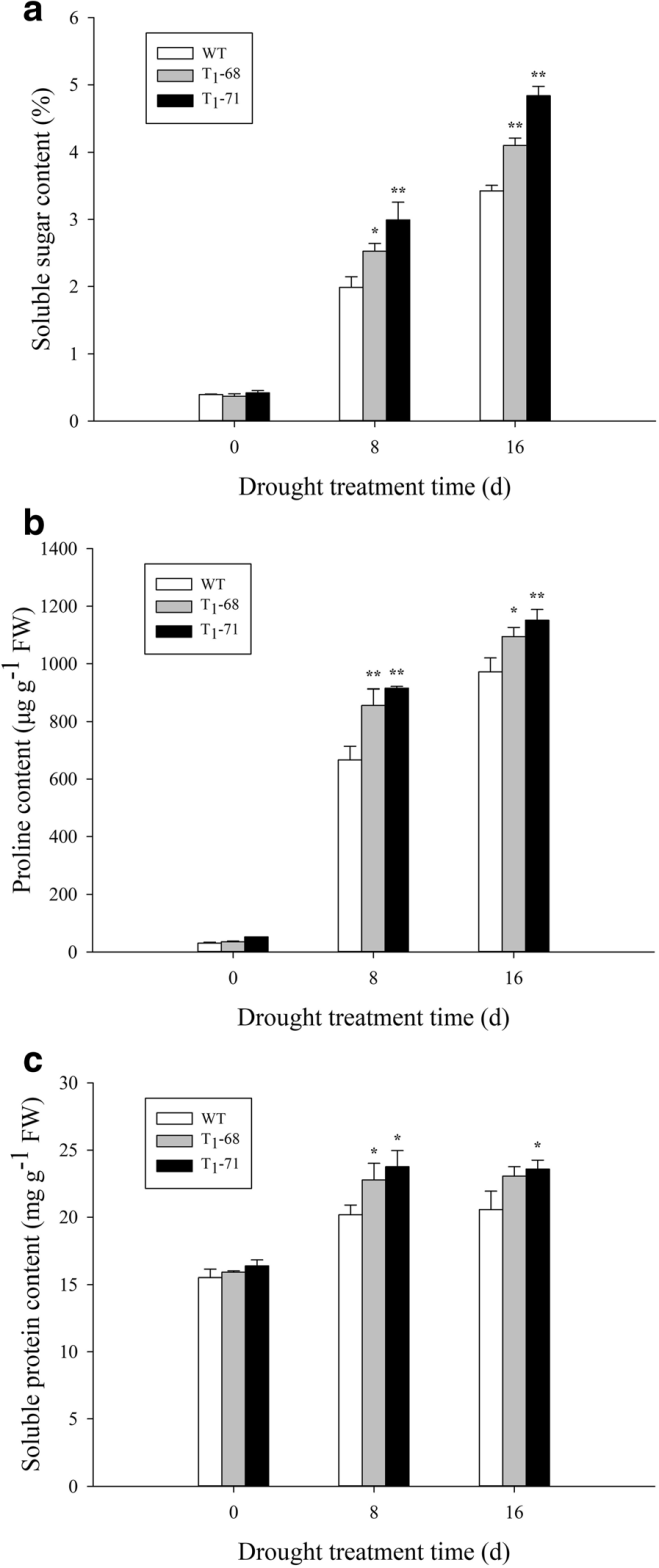
Effects of drought stress on contents of soluble sugar (**a**), proline (**b**) and soluble protein (**c**) in leaves of both wild type (WT) and transgenic tobacco after treatment of 0, 8 and 16 d. Values are means ± SD (*n* = 3). * and ** Significant at *P* < 0.05 and *P* < 0.01 compared with WT, respectively

There were no significant differences between soluble sugar, proline and soluble protein contents in leaves of the WT and OE lines at 0 day (Fig. 6). While with the increasing of treatment time, the soluble sugar and proline content gradually increased and finally reached the peak after 16 days of treatment. Unlike this, the soluble protein content reached the peak at 8 days, and decreased slightly at 16 days. The soluble sugar content in leaves of WT, ‘T_1_–68’, and ‘T_1_–71’ at 16 days increased 8.70, 11.08, and 11.52 times, respectively (Fig. 6a), compared with those at 0 day. The proline content of the ‘T_1_–68’ and ‘T_1_–71’ lines increased by 12.6% (*P* < 0.05) and 18.5% (*P* < 0.01), respectively (Fig. 6b), compared with that of WT. After 8 days of treatment, the soluble protein content of the WT, ‘T_1_–68’, and ‘T_1_–71’ increased by 30.0, 43.2, and 45.2%, respectively (Fig. 6c), compared with that of 0 day. These results indicated that the osmolytes were significantly upregulated by the *CsPLDα* regulation.

### *CsPLDα* expression improved tolerance to drought in tobacco

**Fig. 7:**
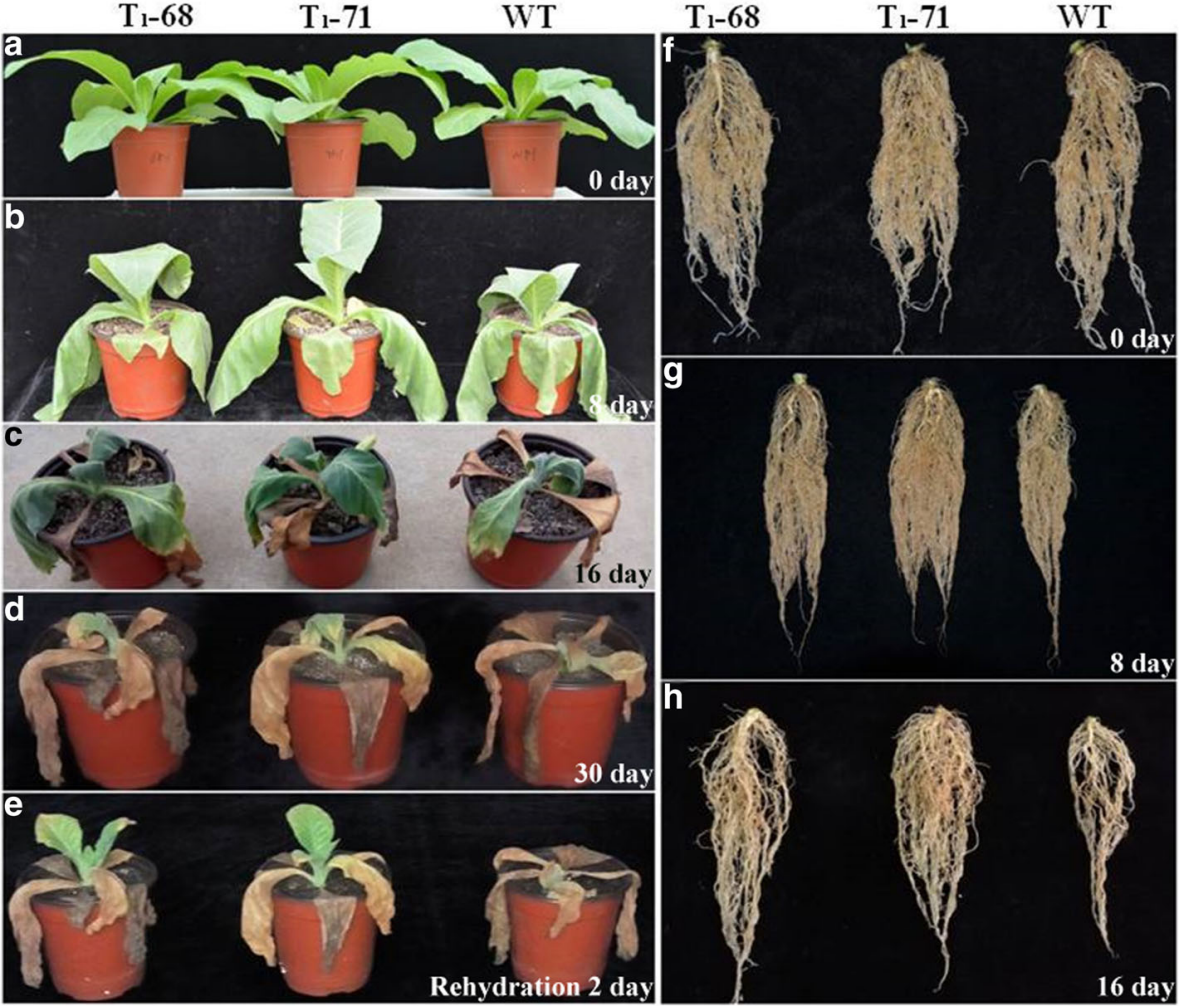
Assessment of drought stress in wild type (WT) and transgenic tobacco seedlings and roots. Phenotypes of WT and transgenic seedlings and roots under conditions of control and drought stress. Photographs were taken after drought stress treatment of 0 d (**a**, **f**), 8 d (**b**, **g**), 16 d (**c**, **h**), 30 d (**d**) and seedlings (e) after refresh treatment of 2 d

**Fig. 8:**
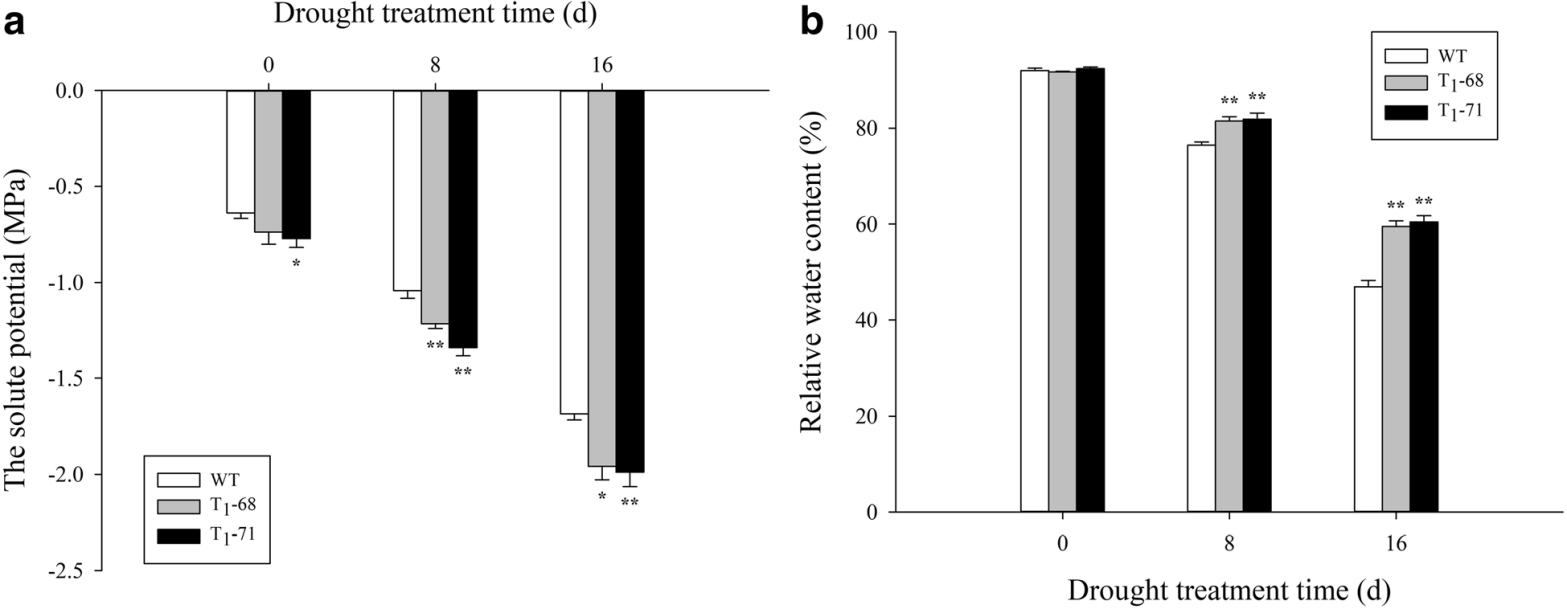
Effects of drought stress on solute potential (**a**) and relative water content (RWC) (**b**) in leaves of both wild type (WT) and transgenic tobacco after treatment of 0, 8 and 16d. Values are means ± SD (*n* = 3). * and ** Significant at *P* < 0.05 and *P* < 0.01 compared with WT, respectively

The Fig. 7 showed that the *CsPLDα* overexpression in tobacco significantly enhanced the tolerance of seedlings to drougth stress after 0, 8, and 16 days of treatment (shoot in Fig. 7a, b, and c and root in Fig. 7f, g, and h). After 30 days of treatment, all lines exhibited severe wilting (Fig. 7d). However, after 2 d of re-watering, the transgenic lines recovered to grow, whereas the WT plants continued to wilt and died (Fig. 7e). The solute potential (*Ψ*s) and RWC were determined to understand the higher tolerance of the transgenic plants to drought than the WT. The *Ψ*s value was more negative in the transgenic plants than in the WT (Fig. 8a). This finding indicated that the transgenic plants possessed a greater potential to retain water than the WT plants. This result was verified by the RWC assay (Fig. 8b). After 8 days of drought stress, the RWC decreased by 16.9% in the WT plants but only by 11.1 and 11.3% in the ‘T_1_–68’ and ‘T_1_–71’, respectively. Similarly, after 16 days of drought stress, the RWC declined by 48.9% in the WT plants but only by 35.1 and 34.6% in ‘T_1_–68’ and ‘T_1_–71’, respectively. Therefore, the *CsPLDα* overexpression enhanced the capacity for osmotic adjustment and consequently increased the water retention during –the drought stress in the transgenic tobacco plants.

## Discussion

This study showed that the *CsPLDα* overexpression exerted positive effects to the plant response to water deficit. When external water is lacking, the water inside the plant cell leaks to the outside of the cell, decreasing cell turgor pressure and changing the osmotic potential across the cell membrane. According to the osmosensor hypothesis, a change in osmotic potential leads to a structural change of the kind of protein in cell membranes called osmosensors; *reduced hyperosmolality-induced [Ca*^*2+*^*]*_*i*_ *increase 1* (*OSCA1)* may be an osmosensor in *Arabidopsis* and is induced by a stimulus in plants [35]. The main osmosensor in plants is a two-component system that includes histidine protein kinase and response regulator protein, and this system plays an important role in the rapid acceptance and transduction to osmotic signal [36, 37]. Aquaporins, one of the most important types of transmembrane osmosensors, can change the structure across the membrane by sensing differences in extracellular water potential and transfer the water potential signal into other intracellular signaling molecules [38]. Through this process, the plant cell receives and conducts the drought signal through various pathways. The regulation of cell membrane lipid modification by phospholipase is one of the important pathways. Such modification can induce cells to produce different kinds of signaling molecules, such as PA, diacylglycerol (DAG), DAG-pyrophosphates, lysophosphatide, free fatty acids, and phosphatidylinositol [39–41].

PA plays an important role in the phospholipid signaling pathway and is mainly produced by phospholipase C and PLD [42]. Water deficit can quickly activate PLD [43, 44], and the activated PLD is involved in ABA-mediated stomatal closure, which helps reduce transpiration and prevent water loss, meanwhile, maintains cell turgor pressure to against drought stress [28, 29]. *PLDα1* and PA regulate ABA-induced stomatal closure by depolymerizing microtubules [45]. *PLDδ* is also involved in ABA-induced stomatal closure. *PLDδ* mRNA levels increase in response to severe dehydration [46], and its expression is upregulated by ABA in guard cells [47]. The interaction between *PLDδ* and glyceraldehyde-3-phosphate dehydrogenases in mediating plant response to ABA and water deficits has been well identified [48]. Zhang et al. [26] suggested that *PLDα1* and *PLDδ* are involved in the same signaling pathway activated by ABA. However, Uraji and Murata [49] proposed that *PLDα1* and *PLDδ* demonstrate a cooperative function in ABA-induced stomatal closure. Moreover, *PLDα1*-deficient plants display delayed ABA-promoted leaf senescence [50]. In the current study, *CsPLDα* was overexpressed in the tobacco plants, and the synthesis and metabolism of ABA in both transgenic and WT tobacco under drought treatment were monitored. First, the expression of endogenous *NtPLDα1* exhibited no difference between the WT and transgenic plants, and the expression of the drought-induced marker gene *NtNAC072* was improved in the transgenic plants under drought stress (Fig. 1). This finding suggested that the *CsPLDα* overexpression could enhance the sensitivity of plants to drought stress. The expression of the ABA-related genes was affected, the most important one of which was *NtNCED1*, which is a rate-limiting enzyme in the ABA synthesis pathway. The *NtNCED1* expression was up-regulated by drought stress and was higher in the transgenic plants than in the WT. The expression of *NtAOG*, which mediates ABA degradation, was lower in the transgenic plants than in the WT. Thus, the ABA content significantly accumulated in the transgenic plants (Fig. 2). A gene in the ABA synthetic pathway, *NtSDR*, was not affected by the drought signal (Fig. 2c), and the previous research has supported the same result [16]. Leaf stomatal closure extent was also observed. The stomatal aperture width of the transgenic plants was less than that of the WT (Fig. 3b). Thus, the increased stomatal closure in the transgenic plants reduced the water loss in comparison with the WT and kept relatively high level water content [27]. The data on leaf water loss after a short water deficit (Fig. 3c) and leaf relative water content (Fig. 8b) after prolonged water deficit both supported this finding. These results indicated that *CsPLDα* was involved in the ABA-promoted stomatal closure during water deficit, further reduced the transpiration, and therefore enhanced the water retention.

Second, PA not only mediated the ABA-promoted stomatal closure to resist drought stress. In *Arabidopsis*, PLD can be used as a positive component to promote proline synthesis [51]. Proline is one of the most effective intracellular osmolytes; it can enhance water retention and maintain membrane structure stability and enzyme activity [52, 53]. Therefore, proline content can directly or indirectly reflect the strength of the resistance of plants to osmotic stress. Other osmolytes maintain osmotic balance, including soluble sugar [54, 55] and glycine betaine [56]. Soluble sugar content is increased in enhanced *ZmPLC1* expression in transgenic maize [57]. In the current study, the contents of proline, soluble sugar, and soluble protein in both the transgenic plants and WT accumulated rapidly and enhance the resistance to drought stress. However, in prolonged drought stress, the osmolyte content in the transgenic plant was more than that in the WT (Fig. 6); hence, the solute potential in the transgenic plant was less than that in the WT (Fig. 8a). These results showed that the *CsPLDα*-driven PA could mediate the osmolyte synthesis to maintain the osmotic balance and alleviate the stress damage.

MDA is commonly used to indicate lipid peroxidation and increases in response to short-term drought stress [58, 59]. EL is equally useful in stress [60]. PLD is also involved in membrane lipid remodeling and rearrangement, which contribute to the synthesis of other kinds of lipids under stress, especially in phosphorus deficit, to maintain the balance of membrane system structure and cell function [61–64]. In the current study, the MDA content and EL in the leaves of the transgenic plants were significantly lower than those of the WT (Fig. 5). This finding indicated that the overexpressed *CsPLDα* could reduce the damage of membrane lipid peroxidation, maintain the membrane system structure stability and function, and decrease the membrane ion leakage during water deficit.

**Fig. 9:**
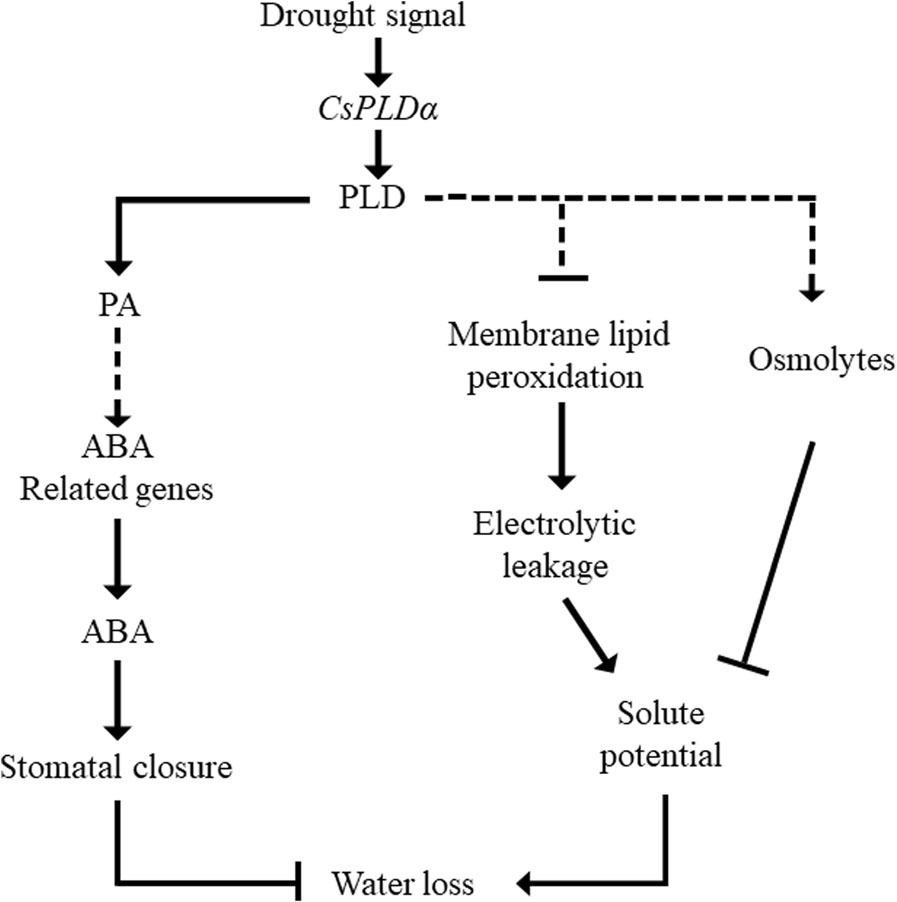
Schematic illustration for main mechanism functioned by *CsPLDα*. 1) *CsPLDa* and CsPLD*α*-produced PA can mediate the stomatal closure by ABA synthesis and metabolism to reduce water loss; 2) *CsPLDα* facilitates the accumulation of osmolytes to reduce water loss; 3) and *CsPLDα* also stabilizes the membrane lipid balance to inhibit ion leakage which led to a higher solute potential and keeps water intracellular

On the basis of the present and previous results, we concluded that the tobacco seedlings with overexpressed *CsPLDα* exhibited improved resistance to water deficit. A schematic illustration for a possible mechanism of *CsPLDα* in plants is presented in Fig. 9. The main adaptive strategies to water deficit are the following: (1) *CsPLDα* and *CsPLDα*-produced PA can decrease water loss by ABA-mediated stomatal closure, and (2) *CsPLDα* facilitates the accumulation of osmoprotective compounds to maintain osmotic balance and stabilize the membrane system. However, previous research showed that plants with overexpressed *PLDα1* display increased susceptibility to drought stress under prolonged water deficit [21]. PA is not conducive to the stability of membrane system, and its presence can accelerate the formation of hexagonal H_II_ phase, break the membrane lipid bilayer structure, and destroy membrane integrity and cell function [65]. PA can be phosphorylated to pyrophosphate DAG, a new type of phospholipid [26]. These reactions may decrease the function of signal transduction. The identification of the upstream regulators and downstream targets that are involved in PLD signaling transduction in plant stress responses will be essential in unraveling the cross-talk between lipid signaling cascades and other signaling pathways. These findings revealed that the role of *CsPLDα* and PA in the regulatory mechanism of plant response to stress is extremely complex. Thus, further studies are needed to verify the interaction between these different functions.

## Conclusions

Both overexpressed *CsPLDα* and it produced-PA are involved in controlling water loss by regulating more stomatal closure and the homeostasis of solute potential, therefore, *CsPLDα* is expressed dominantly in vigorously growing tobacco cells under drought stress, both in leaves and roots, and its overexpression plants can improve the tolerance to water deficit. These also indicated the role of *CsPLDα* in the regulating mechanism of plant response to drought stress is extremely complex, thus we still need further study to verify the interaction between all these different functions.

## Methods

### Plant materials, growth condition, and drought stress treatment

Tobacco (*Nicotiana tabacum* cv. NC89) was used for the assays as the wild type, and the transgenic lines ‘T_1_–68’ and ‘T_1_–71’ resulting from self-fertilization were produced during a previous research [33]. Based on the previous study [33, 34], the WT seeds were sown 2 days earlier, and all seeds were sterilized and sown into plastic plots. Seedlings were grown in a controlled environment with an air temperature of 28 °C (day)/ 23 °C (night), a light intensity of 140 μmol m^− 2^ s^− 1^, and a relative humidity of 60%. The four-week seedlings were transferred into a plastic plug filled with nursery substrate, and grew in glass greenhouse at Shandong Agricultural University. The plants were watered with a Hoagland complete nutrient solution just like describing at [34] before treatment and refreshment. The experiment was conducted under natural conditions with an air temperature of 25–30 °C during the day and 18–25 °C at night. After 2 weeks, the young tobacco seedlings were used as experimental materials for the treatments. The drought stress treatment was performed according to the method described by Hong et al. [21]. The plugs were arranged in a completely randomized block design with 15 replicates per treatment. The tobacco plants were watered with the Hoagland nutrient solution the day prior to the treatment (0 day), and the watering was stopped in the succeeding 30 days. All lines were refreshed for 2 days. The samples were collected after 0, 8, and 16 days of treatments, and quantitative real-time PCR (RT-PCR) were performed. The H_2_O_2_ and ABA contents; plant fresh weight (FW) and dry weight (DW); electrolytic leakage (EL); malondialdehyde (MDA), proline, soluble sugar, and soluble protein contents; relative water content (RWC); and solute potential were measured in the leaves or roots.

### Stomatal closure and water loss determination

To determine the percentage of closed stomata and stomatal aperture in response to dehydration, the leaves of the tobacco seedlings prior to the treatment were detached and exposed to an illuminated incubator with an air temperature of 23 °C and a cool, white light intensity of 125 μmol m^− 2^ s^− 1^. At 0, 10, 20 and 30 min after detachment, the percentage of closed stomata and stomata aperture were determined using a scanning electron microscope (Japanese Electronics Co., Ltd.) and analyzed using the Image-Pro software. Each treatment in each genotype used 30 stomata. Water loss was determined after 0, 0.5, 1, 3, 5, and 8 h.

### Quantitative RT-PCR

Total RNA was extracted from the leaves by using the TRIzol method in accordance to the manufacturer’s instructions (CWBio), and the reverse transcription reaction was done by using the TransScript One-Step gDNA Removal and cDNA Synthesis SuperMix (TransGen). The primer sequences are shown in Additional file 1: Table S1. For the determination of drought stress-associated gene expression, the qRT-PCR was performed using the TransStart® Tip Green qPCR SuperMix, and detected by using an ABI 7500 RT-PCR instrument (Thermo Fisher Scientific, USA), as described in the literature [66]. Each expression profile was independently verified in triplicate. Data were analyzed using the SDS 2.0 software (ABI), and the relative gene expression levels were calculated using the 2^−△△Ct^ method [67].

### Extraction and determination of ABA content

The ABA extraction and determination were performed in accordance with the method of Cheng et al. [16] with modifications. Fresh samples (0.5 g) were homogenized and extracted, then incubated at 4 °C. Phosphate buffer was added to the extract, and 1 ng of ABA (SA8750, Beijing Solarbio Life Sciences, Beijing, China) was added to each sample as internal standard, in accordance with the method of Asami et al. [68]. After distilling the acetone, lipids were removed by partitioning the aqueous concentrate with hexanes. Then the aqueous phase was adjusted to a pH 2.5 and extracted using ethyl acetate. The acidic fraction was dried and dissolved in methanol. The solution was subjected to HPLC on a μBondapak C18 (30 cm × 0.78 cm column; Waters, Milford, MA, USA). The ABA was collected from 10.0 min to 12.0 min. The fractions containing ABA were dried and methylated with diazomethane. The methylated ABA was used for the LC-MS analysis.

The LC-MS analysis was performed using a triple quadrupole liquid chromatography mass spectrometer (TSQ Quantum) with a DB-1 capillary column. The chromatographic conditions were as follows. Thermo Scientific Hypersil C18 column was used with mobile phases of methanol (B) and water (D). In the elution gradient, the mobile phase B was increased from 20 to 90% within 6 min, maintained at 90% for 2 min, and then decreased to 20% within 4 min. The velocity, column temperature, and sample quantity were 0.3 ml/min, 30 °C, and 5 μl, respectively. The mass spectrometry conditions were as follows. The negative ion mode was utilized for electrospray power. The spray voltage, gasification temperature, sheath pressure, auxiliary air pressure, ion transport tube temperature, collision gas, and scanning mode were 3.5 kV, 350 °C, 35 arb, 15 arb, 350 °C, 1.5 mTor, and SRM, respectively.

### Determination of plant FW and DW

Under the stress conditions after 0, 8, and 16 days of treatment, three plants each from the WT, ‘T_1_–68’, and ‘T_1_–71’ were collected. The FWs of tobacco were measured immediately. The samples were dried at 80 °C for 48 h, and then the DWs were measured.

### EL and lipid peroxidation analysis

The EL and MDA content were determined in accordance with the method of Wang et al. [69] with modifications. Leaves were sampled at 0, 8, and 16 days after treatment and washed with deionized water. The membrane ion leakage was expressed as the percentage of initial conductivity versus total conductivity. The MDA content was expressed as the nonspecific absorbance at 600 nm was subtracted from the absorbance at 532 nm, and the difference was used to calculate the amount of MDA by using an extinction coefficient of 155 mM^− 1^ cm^− 1^ [70].

### Determination of proline, soluble sugar, and soluble protein contents

Proline extraction was used an improved sulfo-salicylic acid method which described by Xu et al. [71]. The soluble sugar was determined by using sulfuric acid–anthrone colorimetry [72]. The protein content determination was based on Bradford [73].

### RWC determination

The FWs of the tobacco leaves were measured immediately after detachment from the seedlings. The turgor weight was determined after incubation in deionized water overnight, and the leaf samples were dried at 80 °C for 48 h to obtain the DW. The RWC was calculated based on the following equation: RWC (%) = (FW − DW) / (TW − DW) × 100 [20].

### Measurement of solute potential

The solute potential (*Ψ*s) was measured as described by Gaxiola et al. [74]. The osmotic potential was determined using a vapor pressure osmometer (model 5520, Wescor, USA). *Ψ*s = −moles of solute (*R* × *K*), where *R* = 0.008314 and *K* = 293 **°**.

### Statistical analyses

Values were presented as means ± standard deviations of the triplicates. Statistical analyses were conducted using ANOVA with SAS (SAS Institute, Cary, NC, USA). Differences between treatments were separated by the least significant difference test at *P* < 0.05 and *P* < 0.01.

## Additional file

Additional file 1: **Table S1.** Primers for real-time quantitative PCR. (DOCX 16 kb)

## Abbreviations

ABA: Abscisic acid
CO_2_: Carbon dioxide
DAG: Diacylglycerol
DW: Dry weight
EDTA: Ethylenediaminetetraacetic acid
EL: Electrolytic leakage
FW: Fresh weight
LC-MS: Liquid chromatography mass spectrometer
MDA: Malondialdehyde
NADPH: Nicotinamide adenine dinucleotide phosphate [H]
NO: Nitric Oxide
OE: Overexpression
PA: Phosphatidic acid
PLD: Phospholipase D
qRT-PCR: Quantitative real-time polymerase chain reaction
ROS: Reactive oxygen species
RWC: Relative water content
WT: Wild type
