# Supplementary material for: Cucumber Phospholipase D alpha gene overexpression in tobacco enhanced drought stress tolerance by regulating stomatal closure and lipid peroxidation
Source: BMC Plant Biol. 2018 Dec 14;18:355. doi: 10.1186/s12870-018-1592-y (PMC6293578; doi:10.1186/s12870-018-1592-y)
Supplement: Supplementary file 1 — Table S1. Primers for real-time quantitative PCR. (DOCX 16 kb) [file 12870_2018_1592_MOESM1_ESM.docx]

**Supplementary material**

Table S1 Primers for real-time quantitative PCR

| Primer name | Primer sequence |
| --- | --- |
| *NtNCED1F* | 5'- GATGCCGTGGAAAGTGTCTT-3' |
| *NtNCED1R* | 5'- AGGGAGTGAGTGTCGGGATT -3' |
| *NtAOGF* | 5'- CTCCAGTAAACGCCAATCGT-3' |
| *NtAOGR* | 5'- TCGCAACCTTCTGGTAATCC-3' |
| *NtSDRF* | 5'- TTAGTAGGAGTTGATTCCGAGTT-3' |
| *NtSDRR* | 5'- CGCACTGTGTTACGCATTGT-3' |
| *NtNAC072F* | 5'- TTTGCCACCAGGGTTTCGT-3' |
| *NtNAC072R* | 5'- TGACCTTATCAGTTCCCGTTGC-3' |
| *NtPLDα1F* | 5’- CCATGTTTACGCATCACCAG-3’ |
| *NtPLDα1R* | 5’- CTCCCATCGCAAAGATCAAT-3’ |
| *β-actinF* | 5’- TTAAAGAGAAACTGGCATATGTTG-3’ |
| *β-actinR* | 5’- GCCCATCTGGTAA CTCATAGC-3’ |
